# Transcriptome analysis discloses dysregulated genes in normal appearing tumor-adjacent thyroid tissues from patients with papillary thyroid carcinoma

**DOI:** 10.1038/s41598-021-93526-9

**Published:** 2021-07-08

**Authors:** Huiling He, Sandya Liyanarachchi, Wei Li, Daniel F. Comiskey, Pearlly Yan, Ralf Bundschuh, Altan M. Turkoglu, Pamela Brock, Matthew D. Ringel, Albert de la Chapelle

**Affiliations:** 1 Department of Cancer Biology and Genetics, The Ohio State University, Columbus, OH 43210 USA; 2 Department of Internal Medicine, The Ohio State University, Columbus, OH 43210 USA; 3 Department of Physics, The Ohio State University, Columbus, OH 43210 USA; 4 Department of Chemistry and Biochemistry, The Ohio State University, Columbus, OH 43210 USA; 5 Division of Endocrinology, Diabetes, and Metabolism, Department of Internal Medicine, The Ohio State University, Columbus, OH 43210 USA; 6 The Ohio State University Comprehensive Cancer Center, The Ohio State University, McCampbell Hall South Room 565, 1581 Dodd Drive, Columbus, OH 43210 USA

**Keywords:** Cell biology, Genetics, Pathogenesis

## Abstract

Papillary thyroid carcinoma (PTC) is the most common type of thyroid cancer. The molecular characteristics of histologically normal appearing tissue adjacent to the tumor (NAT) from PTC patients are not well characterized. The aim of this study was to characterize the global gene expression profile of NAT and compare it with those of normal and tumor thyroid tissues. We performed total RNA sequencing with fresh frozen thyroid tissues from a cohort of three categories of samples including NAT, normal thyroid (N), and PTC tumor (T). Transcriptome analysis shows that NAT presents a unique gene expression profile, which was not associated with sex or the presence of lymphocytic thyroiditis. Among the differentially expressed genes (DEGs) of NAT vs N, 256 coding genes and 5 noncoding genes have been reported as cancer genes involved in cell proliferation, apoptosis, and/or tumorigenesis. Bioinformatics analysis with Ingenuity Pathway Analysis software revealed that “Cancer, Organismal Injury and Abnormalities, Cellular Response to Therapeutics, and Cellular Movement” were major dysregulated pathways in the NAT tissues. This study provides improved insight into the complexity of gene expression changes in the thyroid glands of patients with PTC.

## Introduction

Thyroid cancer is the most common type of endocrine cancer. It is estimated that nearly 44,280 Americans will be diagnosed and 2200 will die of the disease in 2021^1^. Papillary thyroid carcinoma (PTC) is the most common form of thyroid cancer, accounting for over 80% of all cases and it occurs about 3 times more often in women than in men^2,3^. Early stage of PTC has an excellent prognosis with an overall 5-year survival rate > 95%; however, later stage of PTC is associated with a poor prognosis. While the underlying factors that result in PTC are incompletely understood, PTC risk is influenced by both environmental and genetic factors^4^. Thyroid radiation exposure during childhood is the most established environmental factor associated with PTC^5^. Lymphocytic thyroiditis (LT), the most common benign thyroid disease, often coexists with PTC, although its role in PTC development is controversial^6–8^. In most studies, obesity has been associated with a higher incidence of thyroid cancer^9,10^. Genetic alterations also play an important role in PTC risk. PTC can occasionally occur in families and a series of GWAS in different populations identified genomic changes that are associated with increased PTC risk^11^. Somatic genetic alterations that cause activation of the MAPK and PI3K-AKT signaling are common in thyroid cancer^12^. Mutations in *BRAF* are particularly common in PTC and can have therapeutic and prognostic implications^13–16^. High-throughput methods have been developed to measure gene expression profiles and identify mutations and fusions to improve PTC diagnosis and treatment^17–20^.

Despite the great advancement in PTC research, the molecular characteristics of histologically normal appearing tissue adjacent to the tumor (NAT) from PTC patients are not well characterized. NAT is commonly used as a control to enable identification of PTC-specific gene expression profiles of coding and noncoding genes^19,21–24^. However, our knowledge of the gene expression profile changes in NAT of patients with PTC versus those without PTC is incomplete. Defining PTC NAT-specific genetic alterations might identify alterations in histologically normal tissue that facilitate PTC oncogenesis and/or progression. For example, we reported previously that overexpression of miR-221 in PTC-associated NAT^21^.

Ria et al. compared gene expression levels of histologically normal thyroid tissues from patients with neoplastic and non-neoplastic thyroid diseases and found twenty-eight genes to be differentially expressed in normal tissues surrounding thyroid cancer; however, tumor tissue was not included in this analysis^20^. Aran et al. compared RNA-seq data sets from heathy tissue samples obtained at autopsy generated as part of the Genotype-Tissue Expression (GTEx) project with NAT tissues from a variety of cancer types analyzed in The Cancer Genome Atlas (TCGA)^25^. They described unique gene expression profiles of NAT in several tumor types, including thyroid cancer^25^. While enabling important comparisons between NAT and normal tissue, postmortem mRNA degradation in autopsy in the normal tissue may introduce important differences from snap frozen NAT^26^. In addition, the impact of the co-existence of LT and patterns of sex-biased gene expression were not addressed^27–31^. While it is not clear whether sex-biased expression is present in NAT, PTC has a marked female sex predisposition suggesting it may be important in this disease^32^. Overall, while useful for PTC diagnostics, the use of NAT as the baseline for comparative gene expression studies may mask early changes preceding the appearance of histologically recognizable tumor that might enable deeper understanding of the requirements for PTC development, more might be influenced by changes in adjacent tissues that occur in response to the tumor. To address this gap in knowledge, we compared gene expression profiles in snap-frozen thyroid tissues of normal thyroid from patients without thyroid cancer, NAT and PTC to identify molecular changes in gene expression unique to the NAT.

## Results

### Evaluation of expression patterns with bulk RNA-seq data

**Figure 1:**
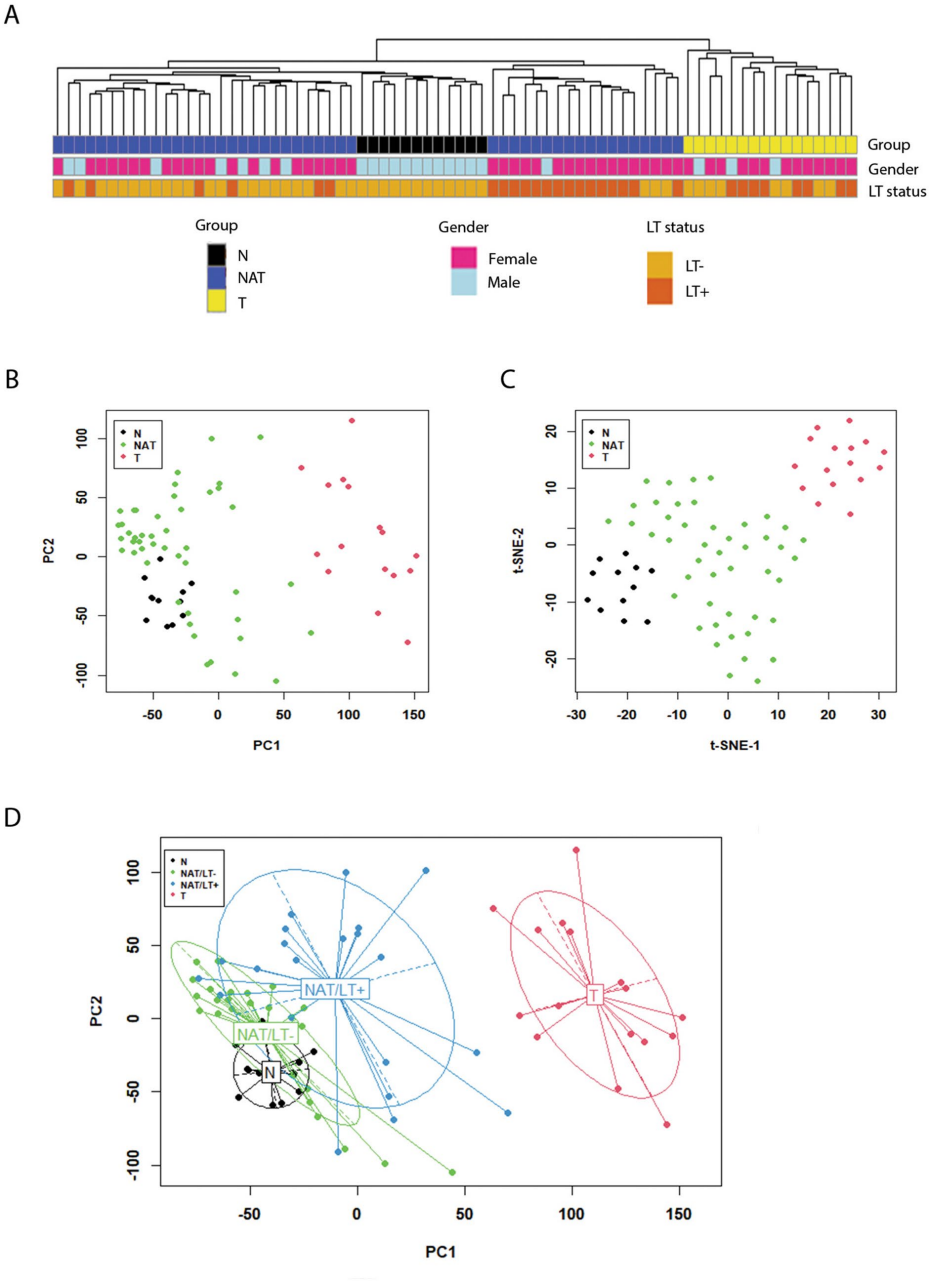
Gene expression patterns and correlation with clinical information. (**A**) Sample clusters. Euclidean distance and average linkage clustering method was used. Gender and the presence of lymphocytic thyroiditis (LT) in each sample are marked with color bars. (**B**) Plot of principal component analysis. (**C**) t-SNE plot. (**D**) Principal component analysis with confidence ellipses according to tissue type. A total of 22,411 genes after filtering were included in the analysis. All the plots were created with log transformed normalized gene expression estimates.

We performed whole transcriptome sequencing (RNA-seq) on three groups of thyroid tissue samples, N (n = 12), NAT (n = 46), and T (n = 16). The T samples were paired with NAT in 16 out of the 46 patients. Deconvolution analysis confirmed a high percentage of thyroid cells in each sample (Supplemental Fig. S1). After filtering genes with zero counts and low expression levels, 22,411 genes were used for analysis. Based on the read counts of these genes, the 74 RNA-seq samples were hierarchically clustered without supervision. The N and NAT samples clustered together, while the T samples clustered as a separate sub-group (Fig. 1A). Samples with co-existing LT on histopathology were identified (NAT/LT+). Among the 21 NAT-PTC/LT+ samples, 15 samples clustered together while 6 samples were scattered with NAT samples without LT (NAT/LT−).

We performed dimensionality reduction analysis to compare the transcriptomes of all 74 samples. Principal component (PCA) analysis and t-Distributed Stochastic Neighbor embedding (t-SNE) plots are shown in Fig. 1B,C. Gene expression patterns tended to correlate with sample groups, with N samples found clustered tightly relative to the other two groups. In general, the NAT samples constituted an intermediate expression state between N and T; the expression profiles of NAT were closer to the N tissue cluster. In addition, the NAT/LT+ and NAT/LT− showed differences in gene expression patterns as seen in the PCA plot (Fig. 1D).

### Differentially expressed genes (DEGs) between NAT and N

We performed DEG analysis comparing NAT and N samples. The cut-off for differential expression was a change of at least 1.5-fold and a BH multiple testing corrected p-value < 0.05 (model was adjusted for age, gender, and LT), and 1000 permutation p-value < 0.05. Ultimately, we obtained 650 DEGs between NAT and N using these criteria.

To assess for possible sex effect on the NAT DEGs, we compared gene expression levels between NAT from females (n = 37) and males (n = 9) and identified 52 sex-different genes (Supplemental Table S2). Notably, 22 (42.3%) sex-different genes were found in the Y chromosome, while 7 (13.5%) genes were in the X chromosome and 23 (44.2%) in autosomes. In contrast, among the DEGs of NAT vs N, the majority (623, 96.3%) were in autosomes, 23 (3.5%) genes were in the X chromosome, and none in the Y chromosome. There were 4 sex-difference genes overlapping with the DEGs of NAT vs N, which were removed for subsequent analysis. Of the final 646 DEGs of NAT vs N, protein coding genes accounted for 273 (42.3%), with 131 (48.0%) upregulated and 142 (52.0%) downregulated. The top 40 coding DEGs are shown in Table 1. The rest of the 373 (57.7%) genes, can be categorized as: pseudogenes (n = 244), lincRNAs (n = 26), snRNA (n = 20), snoRNA (n = 17), antisense (n = 20), processed transcripts (n = 14), sense_intronic (n = 11), misc RNA (n = 11), scaRNA (n = 5), sense_overlapping (n = 2), TEC (n = 2), and retained intron (n = 1). The noncoding genes in the categories of lincRNA, lncRNA, snRNA, snoRNA, and scaRNA are provided in Table 2. Data on the classification and description of gene biotypes can be found in Ensemble (http://useast.ensembl.org/info/genome/genebuild/biotypes.html). The 646 DEGs between NAT and N are provided in Supplemental Table S3.

**Table 1:** Top 40 differentially expressed coding genes between NAT and N^a^.

Gene ID^b^	Gene name	P value^c^	Fold change (NAT/N)	DEGs of NAT/LT− vs N^d^
ENSG00000213977.7	TAX1BP3	2.43E−12	3.67	Yes^e^
ENSG00000147586.9	MRPS28	4.97E−11	2.39	Yes
ENSG00000257727.5	CNPY2	1.34E−08	2.21	Yes
ENSG00000148180.18	GSN	2.1E−08	3.82	Yes
ENSG00000174903.15	RAB1B	4.09E−08	−1.59	Yes
ENSG00000203791.14	METTL10	4.15E−08	1.93	Yes
ENSG00000170296.9	GABARAP	8.02E−08	4.17	Yes
ENSG00000143368.9	SF3B4	7.59E−07	−1.56	Yes
ENSG00000136371.10	MTHFS	2.29E−06	2.42	Yes
ENSG00000171295.12	ZNF440	2.73E−06	1.53	No^e^
ENSG00000088038.17	CNOT3	4.31E−06	−2.16	Yes
ENSG00000132471.11	WBP2	6.74E−06	−1.62	Yes
ENSG00000277203.1	F8A1	8.99E−06	−1.75	Yes
ENSG00000183889.12	AC138969.4	1.44E−05	−1.84	Yes
ENSG00000198171.12	DDRGK1	1.87E−05	−1.54	Yes
ENSG00000128739.21	SNRPN	2.59E−05	2.29	Yes
ENSG00000104969.9	SGTA	2.76E−05	−1.51	Yes
ENSG00000263290.5	SCAMP3	3.07E−05	−1.58	Yes
ENSG00000164039.14	BDH2	3.16E−05	1.71	Yes
ENSG00000147955.16	SIGMAR1	3.26E−05	−1.56	Yes
ENSG00000175274.18	TP53I11	5.42E−05	−1.74	Yes
ENSG00000270011.6	ZNF559-ZNF177	5.71E−05	2.80	Yes
ENSG00000205544.3	TMEM256	6.32E−05	3.71	Yes
ENSG00000111775.2	COX6A1	6.36E−05	2.28	Yes
ENSG00000100350.14	FOXRED2	6.66E−05	−1.87	Yes
ENSG00000279576.1	AP000769.1	8.82E−05	16.16	Yes
ENSG00000181264.8	TMEM136	9.61E−05	1.54	Yes
ENSG00000115239.21	ASB3	9.75E−05	1.69	Yes
ENSG00000100348.9	TXN2	0.000123	−1.57	Yes
ENSG00000167182.13	SP2	0.000132	−1.82	Yes
ENSG00000277462.1	ZNF670	0.000133	2.05	Yes
ENSG00000167644.11	C19orf33	0.000162	19.70	Yes
ENSG00000134590.13	FAM127A	0.000174	−1.62	Yes
ENSG00000239697.10	TNFSF12	0.000177	−1.83	Yes
ENSG00000164898.12	C7orf55	0.000191	3.25	Yes
ENSG00000124614.13	RPS10	0.000237	13.41	Yes
ENSG00000188257.10	PLA2G2A	0.000277	−14.64	Yes
ENSG00000196757.7	ZNF700	0.000284	1.59	Yes
ENSG00000116649.9	SRM	0.000299	−1.83	Yes
ENSG00000189171.14	S100A13	0.000328	1.93	Yes

^a^NAT, normal appearing tumor adjacent tissue; N, normal thyroid control.

^b^The human genome GRC38 was used for gene mapping and annotation.

^c^Benjamini and Hochberg multiple testing corrected p-value. Model was adjusted for age, gender and LT status.

^d^NAT/LT−, normal appearing thyroid tissue without co-existance of lymphocytic thyroiditis.

^e^Yes, the gene is overlapping with the DEGs of NAT/LT− vs N; No, no overlapping.

**Table 2:** Top 40 differentially expressed non-coding genes between NAT and N.

Gene ID	Gene name	Gene biotype	P value	Fold change (NAT/N)	DEGs of NAT/LT− vs N
ENSG00000242299.1	RP11-234A1.1	processed_pseudogene	3.671E−24	8.28	Yes
ENSG00000254911.3	SCARNA9	antisense	1.74E−18	7.48	Yes
ENSG00000233328.3	PFN1P1	processed_pseudogene	1.19E−15	−7.49	Yes
ENSG00000136149.6	RPL13AP25	processed_pseudogene	2.877E−14	7.24	Yes
ENSG00000272779.1	LL22NC03-80A10.6	transcribed_unprocessed_pseudogene	1.681E−13	4.04	Yes
ENSG00000226525.5	RPS7P10	processed_pseudogene	4.401E−13	6.42	Yes
ENSG00000278771.1	Metazoa_SRP	misc_RNA	4.444E−10	3.68	Yes
ENSG00000253954.3	HMGN1P38	processed_pseudogene	7.143E−10	−3.10	Yes
ENSG00000259918.1	NDUFA5P11	processed_pseudogene	4.037E−09	−3.79	Yes
ENSG00000243199.1	RP11-408P14.1	processed_pseudogene	4.037E−09	5.23	Yes
ENSG00000233913.7	CTC-575D19.1	processed_pseudogene	1.224E−08	6.18	Yes
ENSG00000251733.1	SCARNA8	scaRNA	1.336E−08	−6.45	Yes
ENSG00000178464.6	CTD-2192J16.15	processed_pseudogene	1.507E−08	4.98	Yes
ENSG00000256745.1	RP11-680H20.1	processed_pseudogene	2.153E−08	3.88	Yes
ENSG00000235776.2	AC000089.3	processed_pseudogene	2.215E−08	20.89	Yes
ENSG00000259706.1	HSP90B2P	processed_pseudogene	2.254E−08	2.53	Yes
ENSG00000230629.2	RPS23P8	processed_pseudogene	3.119E−08	3.54	Yes
ENSG00000224631.4	RP11-51O6.1	transcribed_processed_pseudogene	4.399E−08	7.42	Yes
ENSG00000231767.3	RP11-92K2.2	processed_pseudogene	5.442E−08	7.41	Yes
ENSG00000272101.2	AC243587.1	processed_pseudogene	1.158E−07	3.74	Yes
ENSG00000236534.1	H3F3BP1	processed_pseudogene	2.069E−07	4.02	Yes
ENSG00000236698.1	EIF1AXP1	processed_pseudogene	2.363E−07	2.61	Yes
ENSG00000274574.1	AC006359.1	snRNA	2.363E−07	−3.56	Yes
ENSG00000212607.1	SNORA3B	snoRNA	2.957E−07	−4.84	Yes
ENSG00000282670.1	AC254944.3	lncRNA	4.208E−07	4.36	Yes
ENSG00000234797.5	RPS3AP6	processed_pseudogene	4.208E−07	5.31	Yes
ENSG00000239470.3	RP11-16F15.2	processed_pseudogene	4.208E−07	6.50	Yes
ENSG00000235174.1	RPL39P3	processed_pseudogene	4.568E−07	3.07	Yes
ENSG00000214389.2	RPS3AP26	processed_pseudogene	7.386E−07	4.28	Yes
ENSG00000174977.8	AC026271.5	processed_pseudogene	7.838E−07	2.76	Yes
ENSG00000243829.1	CTB-33G10.1	processed_pseudogene	1.064E−06	61.76	Yes
ENSG00000256393.1	RPL41P5	processed_pseudogene	1.876E−06	4.31	Yes
ENSG00000253683.1	CTB-79E8.3	processed_pseudogene	2.023E−06	3.24	Yes
ENSG00000178660.6	ARMC10P1	processed_pseudogene	2.181E−06	3.17	Yes
ENSG00000266992.1	DHX40P1	unprocessed_pseudogene	2.205E−06	3.79	Yes
ENSG00000274026.1	FAM27E3	transcribed_processed_pseudogene	2.293E−06	−3.17	Yes
ENSG00000265727.2	RN7SL648P	misc_RNA	4.46E−06	1.97	Yes
ENSG00000198618.5	PPIAP22	processed_pseudogene	4.543E−06	4.31	Yes
ENSG00000220749.4	RPL21P28	processed_pseudogene	4.871E−06	3.83	Yes
ENSG00000283390.1	RP11-134F2.7	processed_pseudogene	4.871E−06	42.91	Yes

The abbreviations, gene annotation, and statistics are the same as described in Table 1.

**Figure 2:**
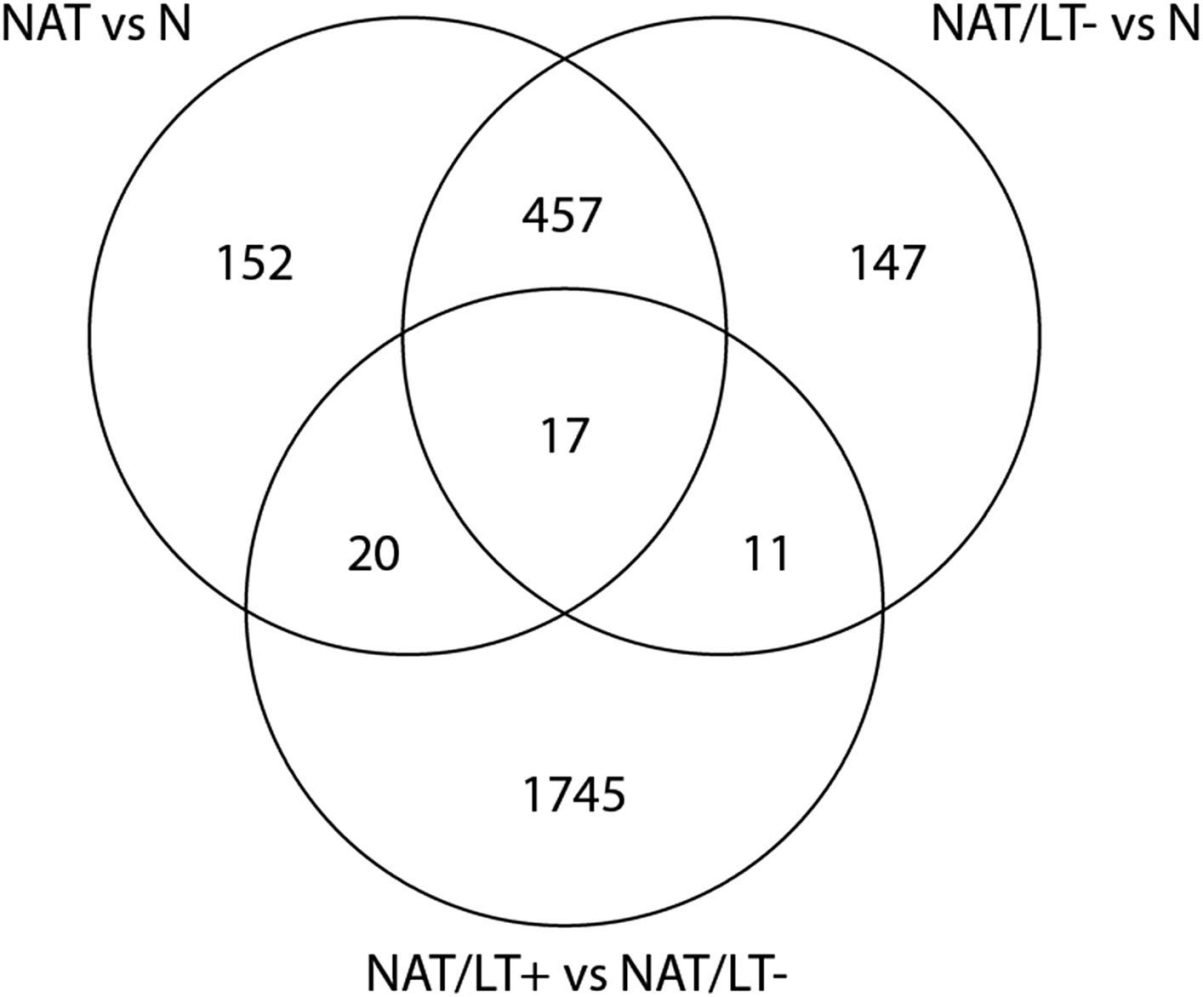
Venn diagram of differentially expressed genes (DEGs) among samples. Venn diagram to illustrate the overlapped DEGs between NAT vs N, NAT/LT− vs N, and NAT/LT+ vs NAT/LT−.

To investigate the impact of LT, we first performed differential gene expression analysis of NAT/LT− vs N and NAT/LT+ vs NAT/LT− using the criteria described above, and obtained 632 DEGs and 1793 DEGs, respectively. The 632 DEGs between NAT/LT− and N are provided in Supplemental Table S4. There are 474 common DEGs between NAT vs N and NAT/LT− vs N (Fig. 2). All the top 40 coding DEGs and the top 40 non-coding DEGs of NAT vs N are present among the DEGs of NAT/LT− vs N except one coding gene (Tables 1, 2). In contrast, there are only 37 common DEGs of all NAT vs N with NAT/LT+ vs NAT/LT− gene list (Fig. 2). Of the 37 LT-related genes, 23 showed opposite directions of gene expression between the two comparisons.

Cho et al. reported aging-related transcriptomic changes in healthy thyroid tissue using the autopsy-derived GTEx dataset^33^. They performed weighted correlation network analysis using all 322 GTEx samples, including 22 LT-positive samples and identified 552 LT-related genes. Of these genes, 456 are included in our annotated 22,411 gene list. Of these 456 genes, 440 (96.5%) are overlapping with the DEGs of NAT/LT+ vs NAT/LT− (Supplemental Table S5).

### DEGs between T and NAT

**Figure 3:**
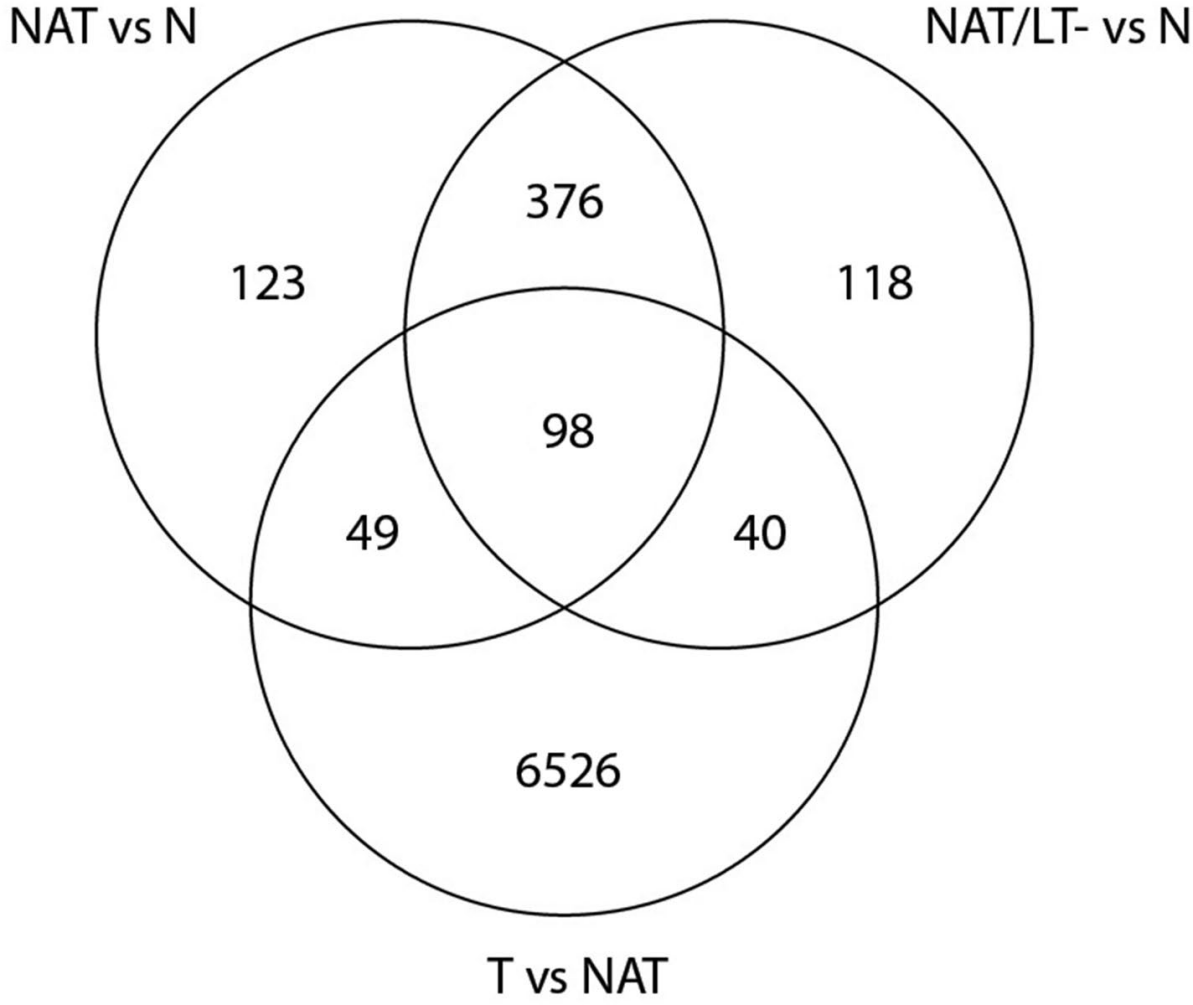
Venn diagram of differentially expressed genes (DEGs) among samples. Venn diagram to illustrate the overlapped DEGs between NAT vs N, NAT/LT− vs N, and T vs NAT.

Using the same approach, we performed pair-wise DEG analysis between T and NAT (n = 16 pairs) and obtained 6,713 DEGs (4,550, 67.8% coding and 2,163, 32.2% non-coding genes). The top 40 DEGs are summarized in Supplemental Table S6. The overlap of the DEGs in NAT vs N, NAT/LT− vs N, and T vs NAT is shown as a Venn diagram in Fig. 3. It is noteworthy that tumor samples yield more dysregulated genes than NAT vs N while only a small number of shared DEGs is observed. We also analyzed the TCGA RNA-seq data of 56 paired (T and NAT) thyroid tissue samples and compared with our data. The DEGs of T vs NAT of the OSU cohort was highly correlated with those of the TCGA data (Supplemental Fig. S2, Supplemental Table S4).

### Dynamic gene expression changes

**Figure 4:**
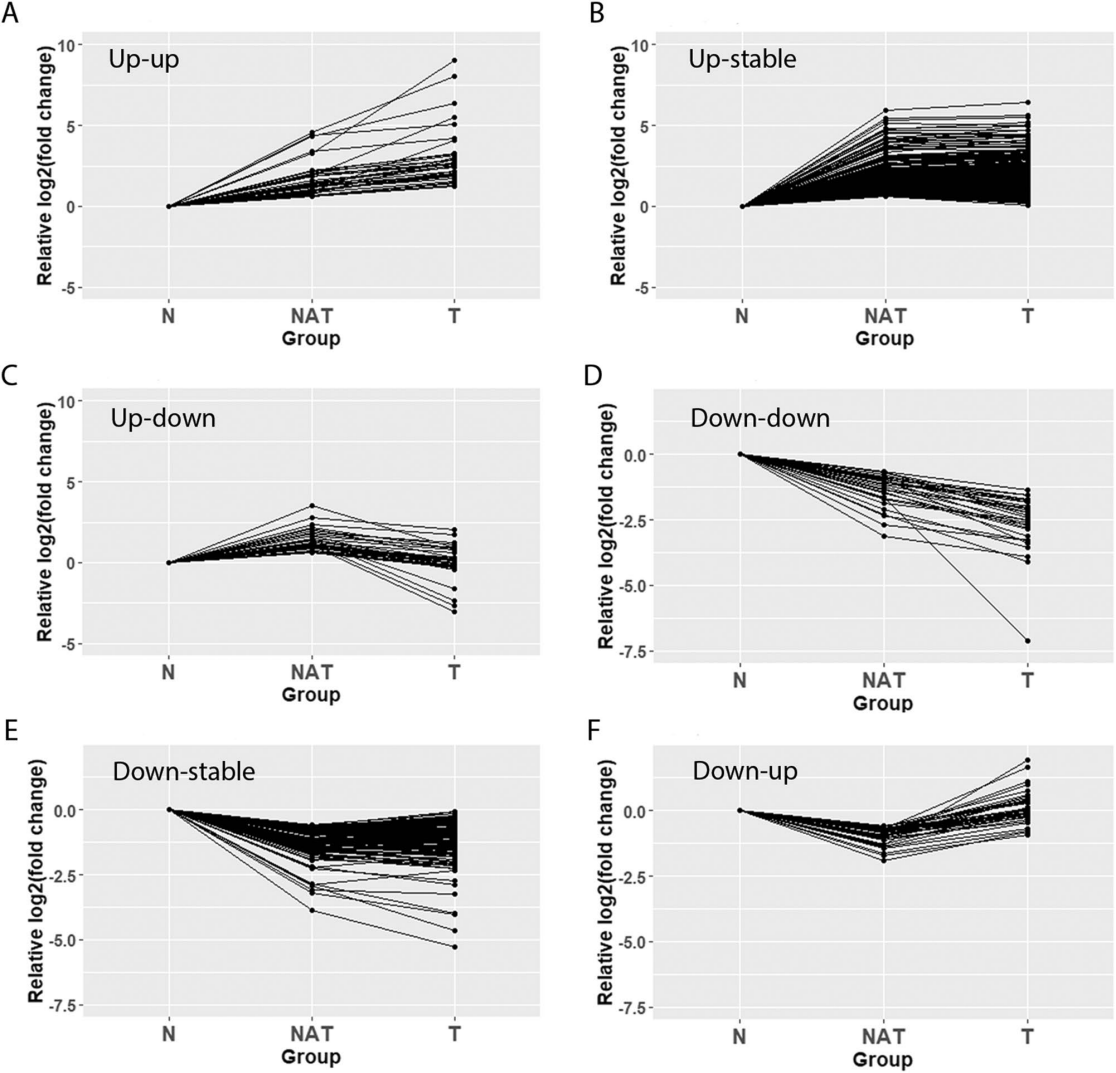
Line plots showing gene expression patterns. Fold changes from N to NAT to T are plotted. (**A**) Up-up, 38 genes. (**B**) Up-stable, 354 genes. (**C**) Up-down, 37 genes. (**D**) Down-down, 31 genes. (**E**) Down-stable, 148 genes. (**F**) Down-up, 38 genes. Up, p-value < 0.05 and log2(fold change) >  = 0.584; Down, p-value < 0.05 and log2(fold change) < −0.584; Stable, not belonging to “Up” or “Down” groups; some stable genes may have relatively high/low fold changes but the p-values are not significant.

Dimensionality reduction analysis showed that the gene expression patterns of NAT samples present an intermediate expression state between N and T. We analyzed the gene expression changes of the 646 DEGs of NAT vs N and their expression changes between T vs NAT. We grouped the expression changes between sample types into six patterns: (1) upregulated from N to NAT to T (up-up, n = 38); (2) up-regulated in NAT, but not changed in T (up-stable, n = 354); (3) up-regulated in NAT, but downregulated in T (up-down, n = 37); (4) downregulated from N to NAT to T (down-down, n = 31); (5) Downregulated in NAT but not changed in T (down-stable, n = 148). (6) Downregulated in NAT but upregulated in T (down-up, n = 38) (Fig. 4, Supplemental Table S2). We performed the similar gene expression pattern analysis with the DEGs of NAT/LT− vs N, and T vs NAT samples without co-existence of LT (T/LT- vs NAT/LT−) as shown in Supplemental Table S4. Among the common DEGs of NAT vs N and NAT/LT− vs N, most of them showed consistent expression patterns (Supplemental Table S7).

### Clustering of DEGs between NAT and N

**Figure 5:**
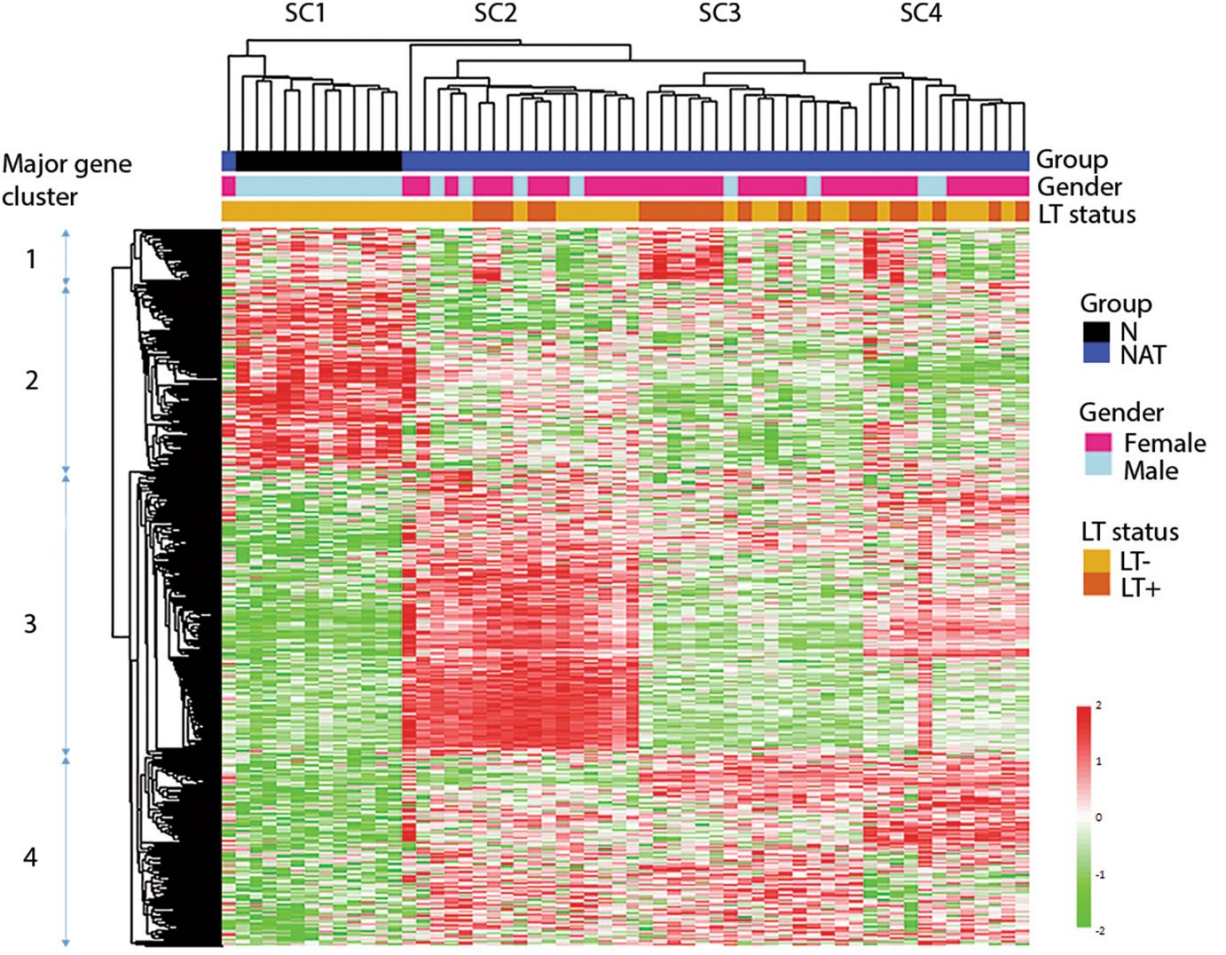
Dendrogram and clusters generated using 646 differentially expressed genes of NAT vs N. The heat map was generated based on 58 samples (46 NAT and 12 N). The annotation bars (above heat map) show sample clusters (SC1-SC4) and gender and the LT status of samples. The bar on the left side demonstrates the range for scaled and centered log2 expression. Red means higher expression and green means lower expression. Four major gene clusters are marked.

To identify groups of patients with similar gene expression and pinpoint co-regulated genes under a subset of samples, we performed hierarchical cluster analysis with the 646 DEGs of NAT vs N, using Euclidean distance and average linkage method as depicted by dendrogram in Fig. 5. Of 58 samples used in the clustering analysis, the 12 N samples and one NAT sample are clustered together into a group labeled SC #1. The rest of the 43 NAT samples are sub-grouped into three major clusters (SCs #2–4), with one outlier not belonging to any of the clusters. The co-existent LT samples (NAT/LT+ samples) are randomly scattered among the SCs #2–4 clusters (Fig. 5). There are 4 major gene clusters. Gene clusters 1 and 2 are largely down-expressed in NAT with variations among samples. Genes in cluster #3 show relatively higher expression in NAT and are mainly enriched in one sample cluster of SC #2. Genes in cluster #4 show relatively higher expression in NAT in three sample clusters of SCs #2–4.

### Annotation of the functions of the DEGs between NAT and N

To explore the functions of the dysregulated genes in NAT, we analyzed related diseases and biological functions using Ingenuity Pathway Analysis (IPA) software. The top five categories of “Disease and Biological Functions” include “cancer, organismal injury and abnormalities, cellular response to therapeutics, cellular movement, and hematological system development and function” (Supplemental Fig. S3). There are 261 genes associated with cancer (Supplemental Table S8). We further examined these cancer genes and found that the great majority of them (256/261, 98.1%) are protein coding genes. The five non-coding cancer genes include one antisense gene, one processed transcript, and three pseudogenes. “Cancer” genes accounted for 88.0% (256 out of 273, 93.8%) of the total protein coding DEGs of NAT vs N. Molecular and cellular functions included “cellular response to therapeutics, cellular movement, cell death and survival, cell morphology, and cell to cell signaling and interaction (Supplemental Table S9). The top 3 molecular networks (Supplemental Fig. S4) are associated with developmental disorder, embryonic development, vitamin and mineral metabolism (network 1), cell signaling, post-translational modification, protein synthesis (network 2), and connective tissue development and function, protein synthesis, tissue morphology (network 3).

## Discussion

In the present study we performed transcriptome analyses with three groups of thyroid tissue samples and characterized their expression profiles. We focused on the dysregulated genes in NAT. Our data suggest that NAT harbors unique molecular changes in gene expression. The majority of dysregulated genes in NAT did not overlap with those in tumors, implying different molecular pathways in these two tissues. We did not observe a significant impact of sex difference in gene expression profile in NAT.

We hypothesized that the unique expression of specific genes in NAT either could indicate early molecular events required for PTC tumorigenesis and/or represent local responses to the primary tumor. Consistent with these hypotheses, the biological functions of the DEGs between NAT and N revealed that about one third of the NAT genes are related to cancer involving cellular functions and maintenance, cell-to-cell signaling and interaction, cell movement, and cellular development. Some of these genes are reported to play a role in cancer. For example, gelsolin (*GSN*) has been found to be dysregulated in various cancers^34–36^. GSN regulates the formation of the actin cytoskeleton^37^, is frequently overexpressed in cancer, and it promotes cell motility^38–40^. *GSN* also is associated with epithelial-mesenchymal transition^41,42^. In our study, *GSN* was overexpressed in NAT and slightly but significantly overexpressed in PTC tumor. Interestingly, in the TR^PV/PV^ mouse model of thyroid cancer, gelsolin is functionally important in Akt-dependent cancer progression, suggesting a functional role for this particular gene in thyroid cancer biology^35,36^.

We observed dysregulated expression of a set of small RNA molecules (scaRNA, snoRNA and snRNA) in NAT versus normal thyroid controls. Interestingly, there were few identified in PTC, consistent with analysis of TCGA^43^. Emerging evidence has revealed the potential significance of snoRNAs and snRNAs in oncogenesis^44,45^. For example, SNORD78 (C/D box) is overexpressed in non-small cell lung and prostate cancer^46^. SNORD50A/B (C/D box), which directly binds and inhibits K-Ras, is deleted across multiple cancer types^47^. Small nuclear RNAs have been incompletely studied in thyroid cancer^48^. Our results suggest that additional functional studies are needed.

PTC is reportedly more common among patients who suffered from LT in some, but not all, studies^7,49^. We found a group of genes preferentially dysregulated in NAT from patients with PTC co-existing with LT. These genes are largely overlapping with LT-related genes in thyroid tissue samples from individuals without thyroid cancer, likely reflective of the underlying LT rather than thyroid cancer^33^. Overall, our data suggest that the differential gene expression in NAT was not driven by LT. The dysregulated genes in NAT largely related to cancer rather than LT. Among the DEGs of NAT vs N, there was a small set of genes showing either continuous upregulation (up-up pattern) from N to NAT to T, or continuous downregulation (down-down pattern) from N to NAT to T, suggesting they might be candidate genes involved in early molecular events in thyroid tumorigenesis. Further validation work is warranted, along with functional studies.

In summary, we address a key gap in the understanding of the molecular underpinning of PTC by comprehensively characterizing differentially expressed genes in normal-appearing tumor-adjacent thyroid tissue from PTC patients using fresh frozen tissue samples,. These results provide a basis for further functional studies defining the earliest tissue requirement that enable PTC development and/or unique events that occur in the histologically normal tissue adjacent to the primary tumor. Additional research is required to determine the roles of the identified genes and processes in PTC tumorigenesis and progression to determine their potential roles as biomarkers and/or treatment targets.

## Materials and methods

Patients and sample collection. The study protocol was reviewed and approved by the Institutional Review Board of The Ohio State University (IRB number: 2006C0047) and was performed in accordance with ethical principles for medical research involving human subjects^50^. Informed consent was obtained from all participants and/or their legal guardians about this study. Normal appearing tumor-adjacent thyroid tissue samples (NAT, n = 46) and paired PTC tumor samples (T, n = 16) were obtained from 46 PTC patients undergoing thyroid surgery. The NAT samples were procured from tissues outside of tumors. Twenty-one samples had co-existent lymphocytic thyroiditis (LT) (PTC/LT+) while 25 did not have LT (PTC/LT−). Of the 16 patients with paired tumor samples 8 were LT+. Normal thyroid tissue samples (N, n = 12) were obtained from laryngeal cancer patients without thyroid lesions who had thyroidectomy as part of their cancer surgery. There were no thyroid diseases revealed in the pathology reports of these normal thyroid samples. The tissue samples were snap-frozen in liquid nitrogen and stored at −80 $^\circ$C. Clinical information is in Supplemental Table S1.

### RNA isolation and quality assessment

Total RNA was isolated using TRIzol reagent (Invitrogen) according to the manufacturer's instructions. The purity of extracted RNA was measured using a NanoDrop ND-1000 spectrophotometer (NanoDrop Technologies LLC). The concentration was assessed by Qubit 2.0 Fluorometer (Agilent Technologies) using an RNA HS Assay Kit. Samples with RNA integrity number greater than 4 as assessed by a BioAnalyzer (Agilent Technologies) with no visible sign of genomic DNA contamination from the HS Nanochip tracings were used for total RNA library generation.

### Preparation of RNA-seq libraries and RNA sequencing

RNA-seq libraries were prepared using the Illumina TruSeq Stranded Total RNA Sample Prep Kit with Ribo-Zero Gold (catalog #RS-122-2201) according to the manufacturer’s protocol. The sequencing was performed in paired end manner, generating 2X 100 bp paired-end reads using the Illumina HiSeq 2500 system. Pre-alignment data QC were assessed with FastQC. Post-alignment data quality was assessed with an in-house quality control pipeline/database for RNA-seq data^51^. RNA-seq data were trimmed for any adapter sequences using AdapterRemoval^52^.

### Gene expression estimate

RNA-Seq reads were mapped to the human genome (GRCh38p7) using HISAT2 and quantified using the featureCounts in the Subread package for 63,299 Ensemble transcriptome/genes^53,54^. Deconvolution analysis was performed to estimate “normal:tumor” cell fraction for each sample^55^. To eliminate bias due to very low expression, genes were filtered-out if each group had zero read counts for more than 25% of the samples or had average read counts below 10. The relative transcript abundance was measured in normalized counts obtained by the median of the ratios normalization method of DESeq2^56^.

### Dimensional reduction analysis

Dimensional reduction analysis and visualization was performed using principal component (PCA) analysis and t-Distributed Stochastic Neighbor embedding (t-SNE) with the Rtsne (version 0.15) library in R package^57^. PCA is an unsupervised linear dimensionality reduction method while t-SNE is an unsupervised non-linear method that preserves the local structure of the data. PCA plots and t-SNE plots were created with log transformed normalized gene expressions using all remaining genes after filtering.

### Differential expression and computational functional analysis

Differential expression analyses were performed with DESeq2, adjusting for age, gender, and LT. Non-paired comparison of NAT vs N and paired comparison of T vs NAT were performed. The Benjamini & Hochberg (BH) method was used to correct p-values for multiple testing. Furthermore, 1000× permutation analysis of samples creates the distribution of the DESeq2 statistic. To exclude artifactual results due to gender bias, genes that showed a significant sex-difference (p-value < 0.05) between males and females among NAT samples were filtered out. Cluster analyses and heat maps were generated to visualize differentially expressed genes. Network, functional and canonical pathway analyses of differentially expressed genes between NAT and N were performed using Ingenuity Pathway Analysis (IPA) software (Ingenuity Systems Inc, www.ingenuity.com).

### The Cancer Genome Atlas (TCGA) RNA-seq data set

The TCGA HTSeq counts were downloaded from the GDC Data portal (https://portal.gdc.cancer.gov/, accessed on March 2020). The DeSeq2 analysis was performed with 58 pairs of T/NAT.

## Supplementary Information

Supplementary Information 1.

Supplementary Information 2.

Supplementary Information 3.

Supplementary Information 4.

Supplementary Information 5.

Supplementary Information 6.

Supplementary Information 7.

Supplementary Information 8.

Supplementary Information 9.

Supplementary Information 10.

## Data Availability

All publicly available datasets used in this study were referenced in the Methods section. Our RNA-seq data have been deposited in NCBI's Gene Expression Omnibus (GEO) with GEO Series accession number GSE165724.
