# Supplementary material for: Transcriptome analysis discloses dysregulated genes in normal appearing tumor-adjacent thyroid tissues from patients with papillary thyroid carcinoma
Source: Sci Rep. 2021 Jul 8;11:14126. doi: 10.1038/s41598-021-93526-9 (PMC8266864; doi:10.1038/s41598-021-93526-9)
Supplement: Supplementary file 10 — Supplementary Information 10. [file 41598_2021_93526_MOESM10_ESM.docx]

He, et al: Supplemental Figures


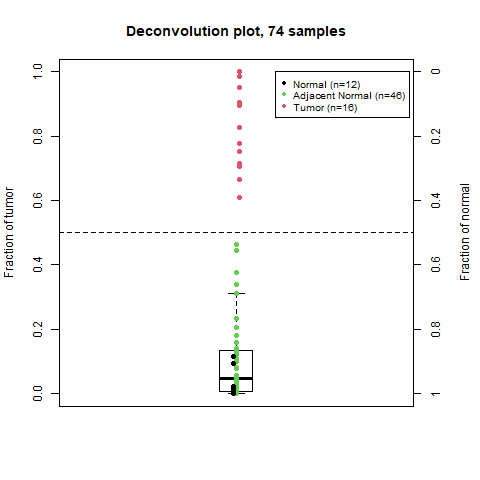


**Supplemental Fig.S1.**  **Deconvolution analysis** **with transcriptome data**. The estimated “normal:tumor” fraction for each sample is shown. The three groups of samples are indicated with color dots. All the 12 N samples are clustered distinctly with >=85% of estimated normal cells. Of the NAT samples, there are 36 samples with >=85% of normal cells and 10 samples with 50-85% of normal cells. There are 9 T samples with >=85% of tumor cells and 7 samples with 50-85% tumor cells.

**Supplemental Fig.S2. Concordance plot to compare DEGs of T vs NAT between OSU data and the Cancer Genome Atlas (TCGA) data.** The TCGA HTSeq counts were downloaded from the GDC Data portal (<https://portal.gdc.cancer.gov/>, accessed on March 2020). The DeSeq2 analysis was performed with 58 pairs of T/NAT. The correlation analysis included a total of 20916 overlapping genes. The correlation is r=0.89 (p-value < 2.2e-16).


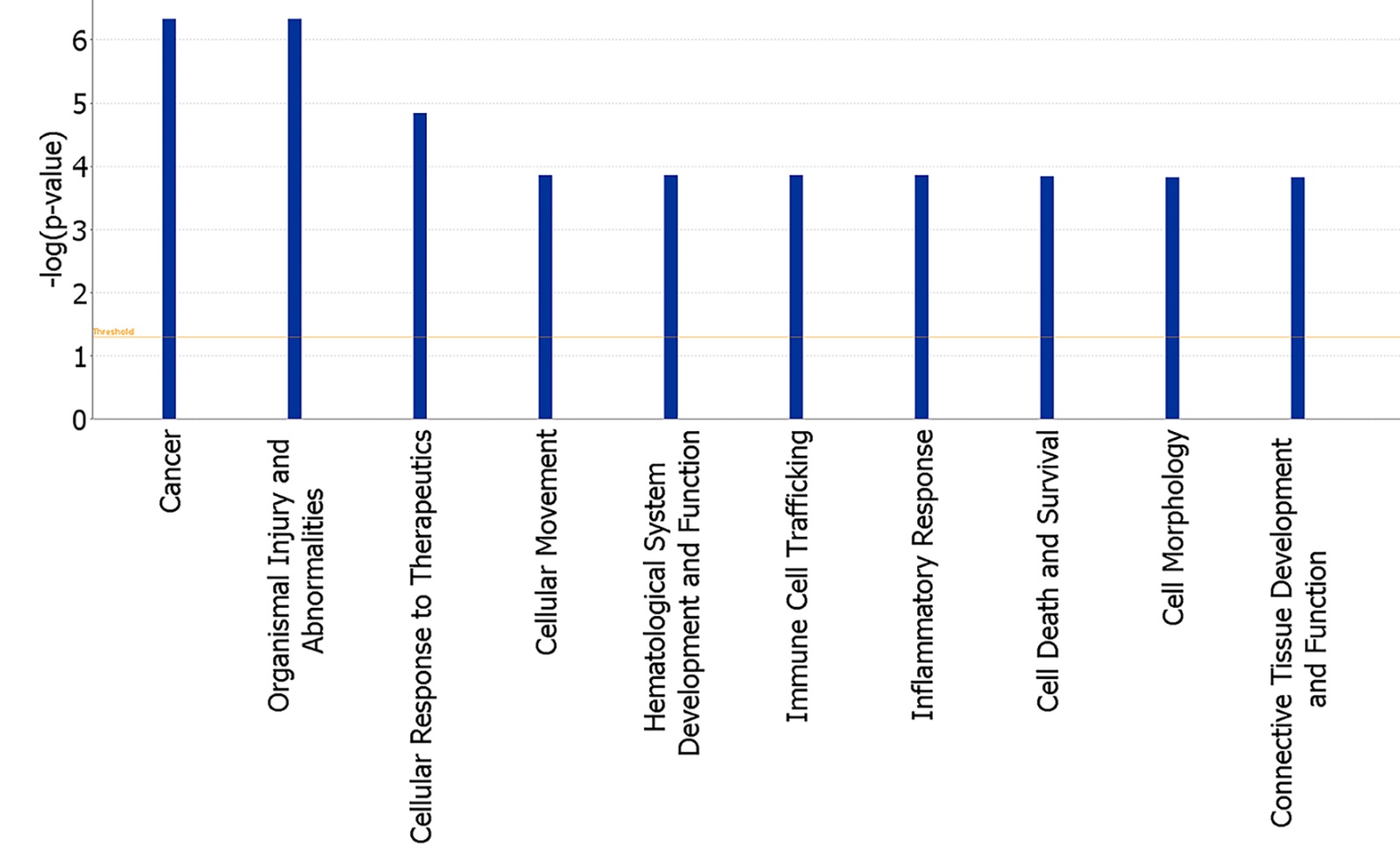


**Supplemental Fig. S3**. **The related diseases and biological functions of the DEGs between NAT and N**. The top ten categories of IPA analysis are shown.


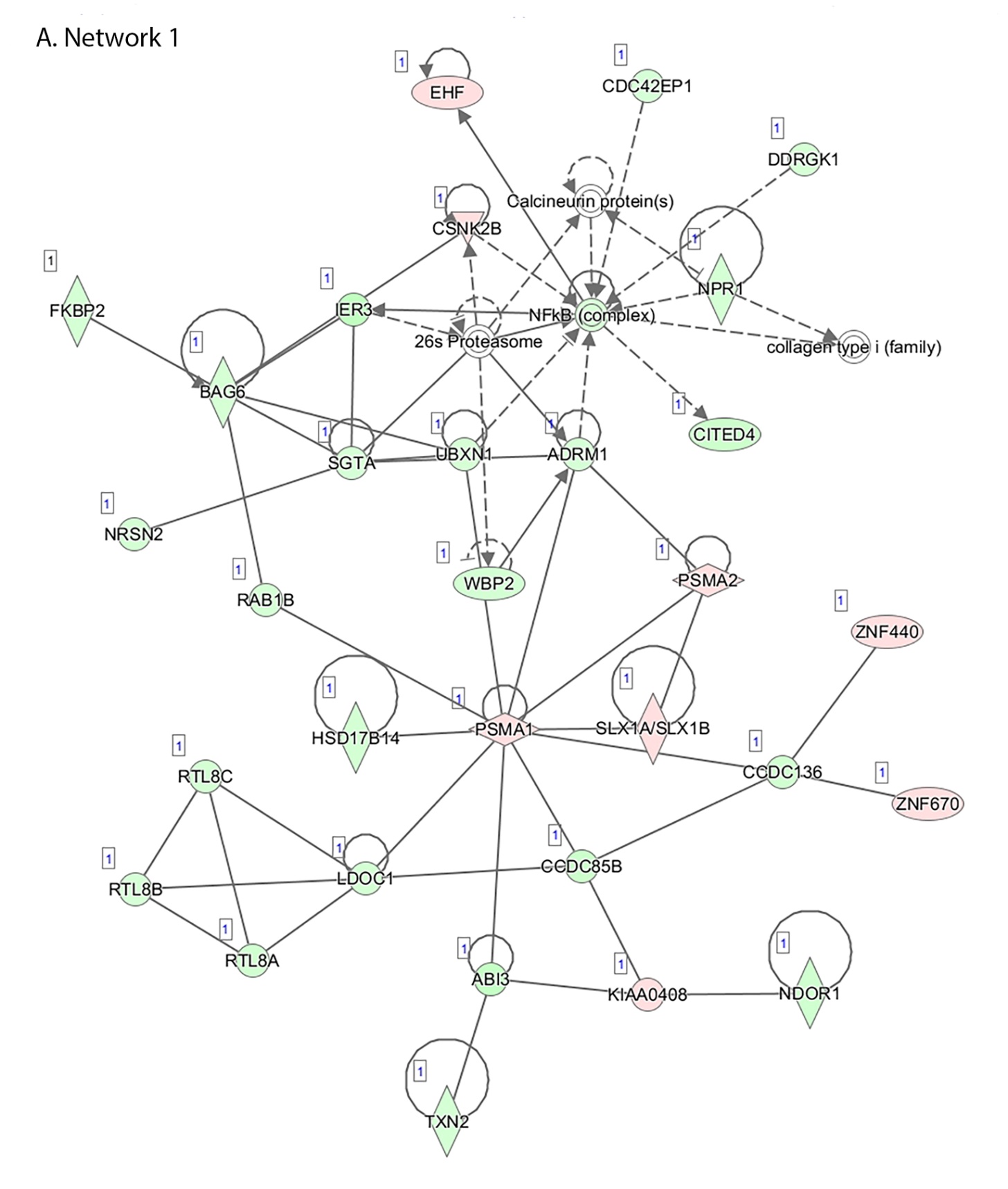


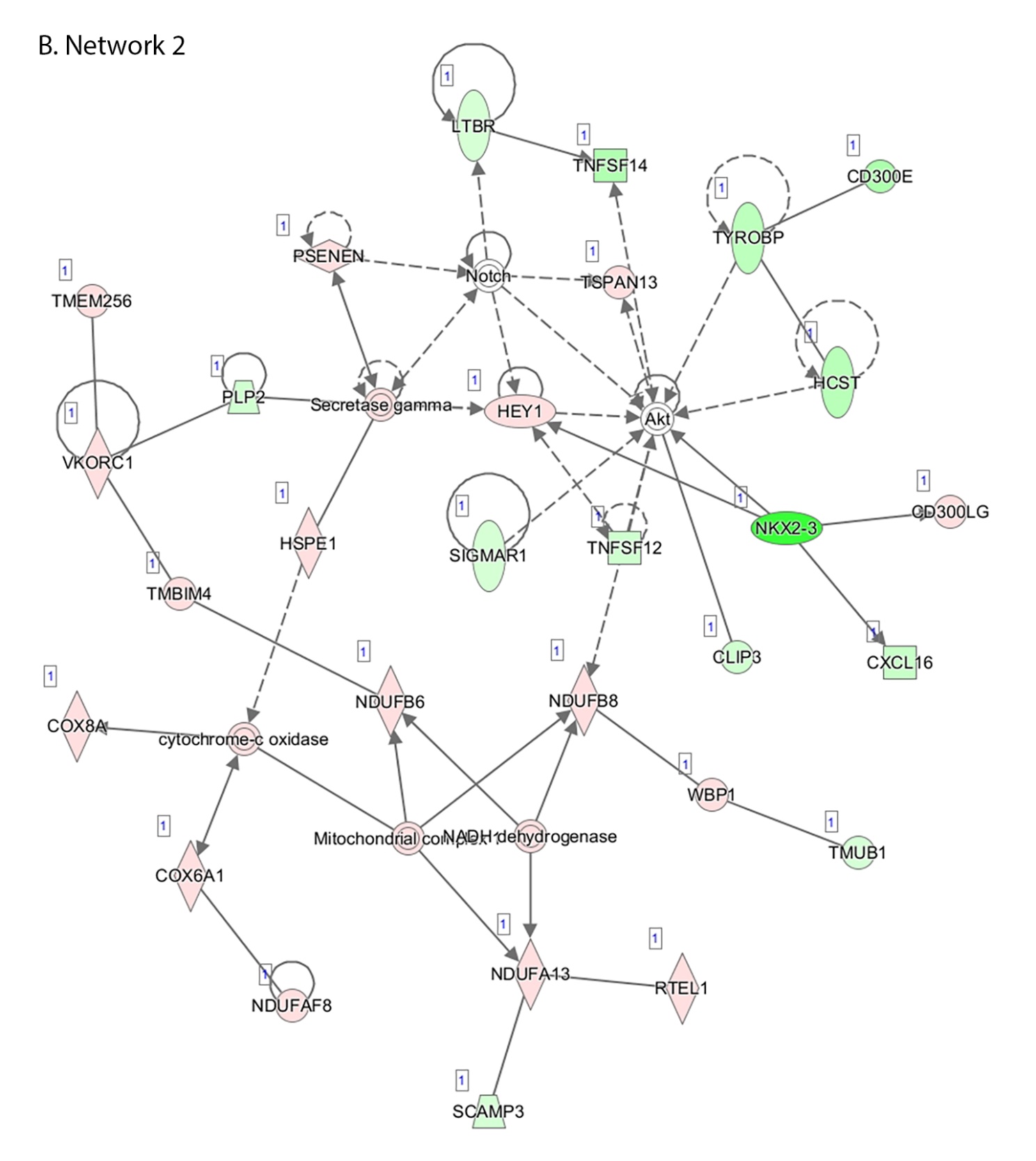


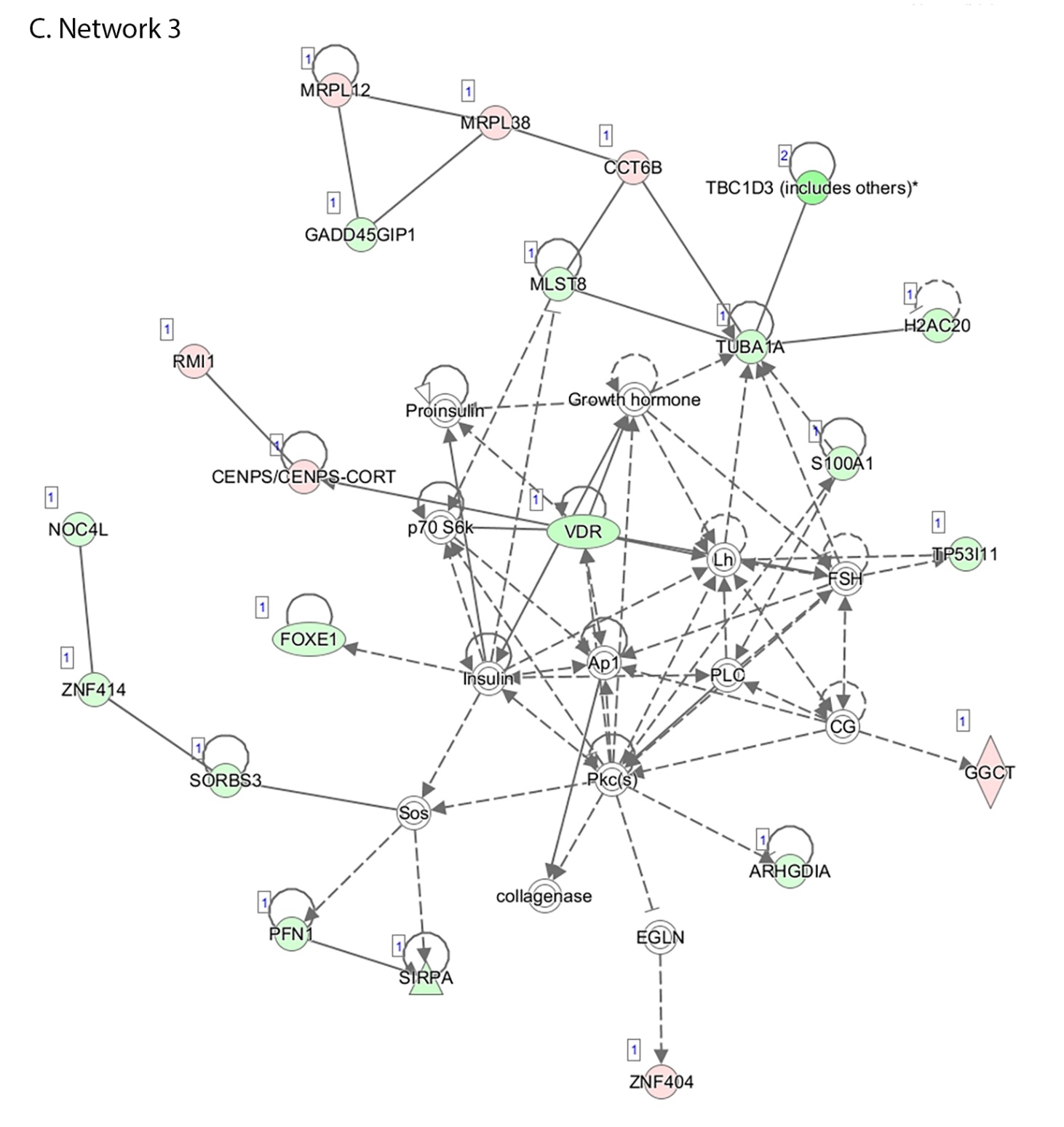


**Supplemental Fig. S4**. **The top 3 molecular networks of the DEGs between NAT and N**. The associated network functions are: (A) Network 1: cell morphology, hematological system development and function, and tissue development; (B) Network 2: cell signaling, post translational modification, and protein synthesis; (C) Network 3: protein synthesis, cell cycle, hair and skin development and function. The networks were obtained by IPA analysis.
